# An invasive ant increases deformed wing virus loads in honey bees

**DOI:** 10.1098/rsbl.2022.0416

**Published:** 2023-01-18

**Authors:** Jana Dobelmann, Antoine Felden, Philip J. Lester

**Affiliations:** ^1^ School of Biological Sciences, Victoria University of Wellington, Wellington 6012, New Zealand; ^2^ Institute of Evolutionary Ecology and Conservation Genomics, Ulm University, Ulm 89081, Germany

**Keywords:** deformed wing virus, Argentine ant, honey bee, invasion, pathogen dynamics

## Abstract

The majority of invasive species are best known for their effects as predators. However, many introduced predators may also be substantial reservoirs for pathogens. Honey bee-associated viruses are found in various arthropod species including invasive ants. We examined how the globally invasive Argentine ant (*Linepithema humile*), which can reach high densities and infest beehives, is associated with pathogen dynamics in honey bees. Viral loads of deformed wing virus (DWV), which has been linked to millions of beehive deaths around the globe, and black queen cell virus significantly increased in bees when invasive ants were present. Microsporidian and trypanosomatid infections, which are more bee-specific, were not affected by ant invasion. The bee virome in autumn revealed that DWV was the predominant virus with the highest infection levels and that no ant-associated viruses were infecting bees. Viral spillback from ants could increase infections in bees. In addition, ant attacks could pose a significant stressor to bee colonies that may affect virus susceptibility. These viral dynamics are a hidden effect of ant pests, which could have a significant impact on disease emergence in this economically important pollinator. Our study highlights a perhaps overlooked effect of species invasions: changes in pathogen dynamics.

## 1. Introduction

Invasive species are perhaps best known for their impacts as predators and competitors, directly driving the decline of species abundance and even extinction [1,2]. However, species introductions can also induce changes in pathogen communities [3] that may play a key role in disease emergence in multi-host multi-pathogen systems [4,5]. For example, protozoan parasites from invasive bumblebees facilitate disease spillover into native South American bumblebees [6] and introduced mink contribute to disease in native British otters via acquiring trematode infections [7]. Such spillover or spillback dynamics in which introduced hosts increase pathogens in local or native species are often overlooked in species invasion [8].

Emerging viral diseases have been linked to losses of a globally important pollinator, the Western honey bee (*Apis mellifera*) [9–11]. Many diseases in bees are caused by positive-sense single-stranded RNA viruses [12], some of which can increase and lead to colony death [10,11]. The deformed wing virus (DWV), for example, is an RNA virus that has been considered to be the primary suspect in the global death of millions of honey bee colonies, and in combination with *Varroa-*mediated transmission, has caused dramatic changes in the viral landscape of honey bees and other arthropods [13–16]. DWV and other bee viruses are not host-specific and have been found in a wide variety of arthropods including spiders, cockroaches and ants [17–20] with active viral replication in some hymenopteran species [13,21]. Invasive wasps, for instance, acquire DWV through preying on honey bees [16]. Similarly, preying on brood, scavenging on dead bees, or robbing honey could expose invasive ants to viruses hosted by bees [22,23] and lead to bees being exposed to pathogens hosted by ants. At least five common bee viruses have been detected in ant species, including DWV and black queen cell virus (BQCV) [18,24–28].

The Argentine ant (*Linepithema humile*) is a globally distributed invasive species that forms dense supercolonies containing many millions of individuals [29,30]. Their invasion can substantially alter recipient communities, with the mechanisms for biotic change historically attributed to competition, predation and the formation of mutualistic interactions [30–32]. Here we aim to test whether Argentine ants disrupt pathogen infections in recipient communities. Using honey bees as a study system, we demonstrate that invasive Argentine ants, which were first observed in New Zealand in 1990 [33], can exacerbate pathogen infections in recipient communities.

## 2. Material and methods

### (a) Experimental set-up and sampling

In the austral summer of January 2019, 18 beehives from an Argentine ant-free apiary were moved into six sites in the Northland region, New Zealand, half with known Argentine ant incursions, placing three hives in each site (electronic supplementary material, figure S1). Ant species presence was established by laying out sugar baits for 30 min in a four-by-five grid, each bait 1 m apart. *Ochetellus glaber* and *Nylanderia* sp*.* ants were found in low numbers in sites without Argentine ants, while no other ant species were observed when Argentine ants were present. Monthly collections of adult worker bees from brood frames took place from January until August, except July. Ants were sampled from foraging trails on beehives. All samples were immediately frozen in a liquid nitrogen dry shipper (Taylor-Wharton, USA). *Varroa destructor* populations were controlled in May or June using Bayvarol® (Bayer New Zealand Limited, New Zealand) and stayed below one mite per 100 bees throughout the experiment. Returning forager bees were counted as an indicator for foraging effort (details in the electronic supplementary material, M1).

### (b) RNA extraction, reverse transcription and pathogen quantification

Ant and bee samples were washed with 10 ml and 500 µl phosphate-buffered saline (pH 7.4, Ambion, Texas, USA), respectively, to reduce the detection of surface-contaminating viruses. Samples of 25 bees (except 12 in January and 20 in February) or 50 ants were homogenized in a bead mill (Precellys Evolution, Bertin Technologies, France). A 0.5 g sample of bee homogenate was added to 600 µl TRIzol® Reagent (Life Technologies, California, USA), while ants were directly homogenized in 500 µl TRIzol® Reagent. Volumes of TRIzol® homogenates used in RNA extractions were 350 µl for bees and 450 µl for ants. We used the Direct-zol RNA Miniprep Plus Kit (Zymo Research, California, USA) including DNase digestion. RNA was eluted in 100 µl nuclease-free water and measured on a NanoPhotometer® (NP80, Implen, Germany). Reverse transcriptions included 1 µg bee-RNA using the High-Capacity cDNA Reverse Transcription Kit (Applied Biosystems, Invitrogen, California, USA) and 800 ng ant-RNA using SuperScript IV (Invitrogen, Thermo Fisher Scientific, USA).

Quantitative PCR (qPCR) was used to examine BQCV, DWV, and Kashmir bee virus (KBV), which are the common viruses widely observed in bees in New Zealand [34], *Nosema ceranae* and *Lotmaria passim* infections in bees with *Apis mellifera actin-related protein 1* (GenBank accession: NM_001185145) was used for pathogen titre normalization (primers in the electronic supplementary material, table S1). Duplicates reactions were run using PowerUp™ SYBR™ Green Master Mix (Applied Biosystems, ThermoFisher Scientific, USA) in a 20 µl volume containing approximately 40 ng complementary DNA on a StepOne™ Real-Time PCR cycler (Applied Biosystems, ThermoFisher Scientific, USA). Cycling followed the fast-cycling mode, including a melt curve stage to confirm single product amplification (electronic supplementary material, M2). Cycle quantification (*C*_q_) values above 35 were excluded to avoid false detection from late-cycle amplification. In ants, TaqMan™ Array Cards with the TaqMan Fast Advanced Master Mix on a QuantStudio 7 Real-Time PCR System (all Applied Biosystems, ThermoFisher Scientific, USA) were used to examine BQCV, KBV and DWV in ants, and normalized against *Dynactin subunit 4* (GenBank accession: LOC105671957). Pathogen load was calculated as 2^−ΔCq^ [35]. Samples were collected six months after hives had been moved (June) and were used in Illumina 100 bp paired-end RNA-seq to compare beehive viromes with and without Argentine ants (electronic supplementary material, S3).

### (c) Statistical analyses

Hive survival was examined using a log-rank test and Kaplan–Meier survival curves using the package *survminer* in R v3.6.3 [36,37]. Infections and bee foraging activity were analysed using permutational multi-variate analysis of variance (PERMANOVA) in the *vegan* package with Manhattan distances [38]. Pathogen load or the number of returning foragers was used as the response variable. Treatment *T* (ants versus no ants), time *M* (month), treatment–time interaction *T × M,* and site *S* nested within treatment were fixed effects. Analyses were run with 999 permutations and restricted to time points to account for repeatedly measuring the same hives [37].

## 3. Results

### (a) Beehive survival and foraging activity

**Figure 1.**
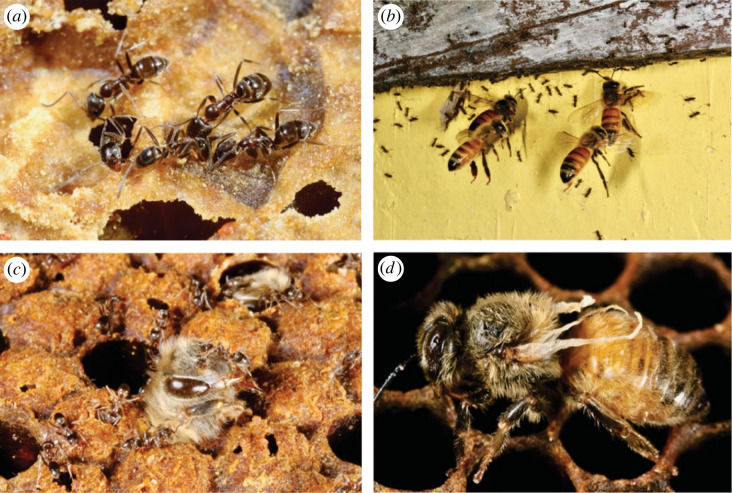
(*a*) Argentine ants foraging inside a beehive. (*b*) Bees hovering over ants that are entering a hive. (*c*) Ants surround a young adult bee while emerging from its cell. (*d*) A bee suffering from the DWV. Photo credits: Phil Lester.

In two out of three sites with Argentine ants, ants foraged on beehives within less than 6 h after the hives were placed. In the third site, we found ants on beehives from March onwards. We observed ants robbing food stores, attacking the bee brood and scavenging on dead adult bees. Bees appeared distressed (figure 1). The three control sites remained Argentine ant-free throughout the experiment. Hive mortality was not statistically affected by ant presence (log-rank test: *p =* 0.878; electronic supplementary material, figure S2); however, only six hives out of 18 survived until late winter in August. Two hives were empty by February; we observed many ants inside both hives in January, which indicates that bees absconded (as observed in [39]). *Varroa* levels did not differ between treatments (PERMANOVA: *F* = 0.001, *R*^2^ = 0.00002, *p* = 0.926) but bee foraging effort was 1.6-fold higher in hives with Argentine ants (*F* = 6.206, *R*^2^ = 0.050, *p* = 0.016; details in the electronic supplementary material, M1 and figure S3).

### (b) Pathogen infections

**Figure 2.**
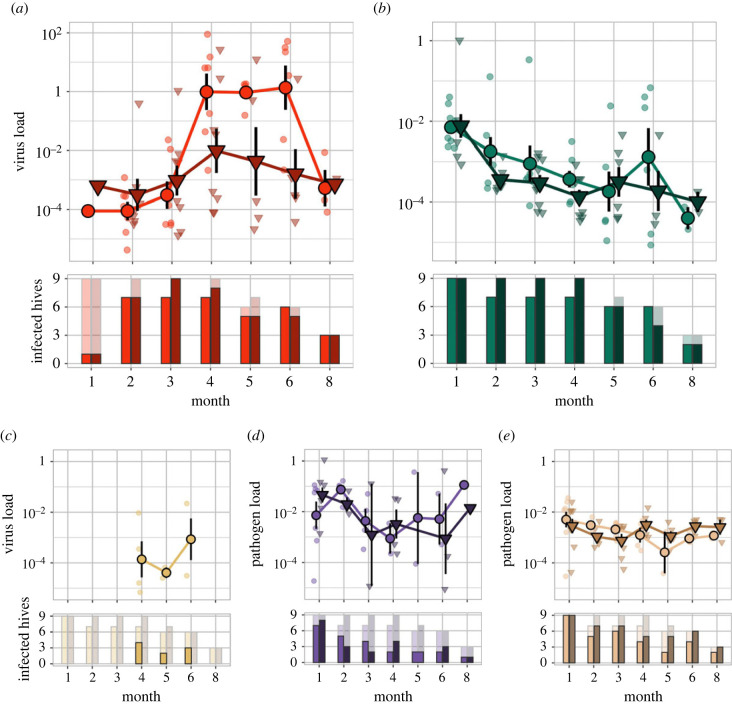
Pathogen titres and prevalence in honey bees with and without Argentine ants. Each pathogen has a top and bottom panel. Top: virus or pathogen loads in beehives with (circles, light colour) and without ants (triangles, dark colour). Large symbols indicate the mean ± s.e. Small symbols are individual data points. Note the different scale for DWV (*a*), BQCV (*b*), KBV (*c*), *Nosema ceranae* (*d*), and *Lotmaria passim* (*e*). Bottom: pathogen prevalence; bars indicate the number of infected beehives with (left, light colour) and without ants (right, dark colour). Transparent bars in the background indicate how many hives were alive at the time point.

DWV titres in bees differed significantly between treatments (PERMANOVA: *F_T_* = 4.653, $R_{T}^{2}=0.046$, *p_T_* = 0.026) and showed strong seasonal variation (figure 2*a*). In sites invaded by Argentine ants, DWV in bees reached extremely high levels between autumn and early winter compared to sites without these ants (figure 2*a*). Viral titres also differed between sites and sampling months (*F_S_* = 43.324, $R_{S}^{2}=0.131$, *p_S_* = 0.007, *F_M_* = 2.324, $R_{M}^{2}=0.114$, *p_M_* = 0.006) with no significant treatment–time interaction (*F_T × M_* = 1.837, $R_{T\times M}^{2}=0.090$, *p_T × M_* = 0.074). BQCV levels were highest in summer and decreased towards autumn and winter (figure 2*b*). Overall, BQCV titres were higher in bees when Argentine ants were present (*F_T_* = 3.420, $R_{T}^{2}=0.044$, *p_T_* = 0.004). Viral titres were also affected by time (*F_M_* = 0.578, $R_{M}^{2}=0.037$, *p_M_* = 0.046), but not by the treatment–time interaction or sampling site (*F_T × M_* = 0.708, $R_{T\times Mn}^{2}=0.045$, *p_T × M_* = 0.652; *F_S_* = 1.382, $R_{S}^{2}=0.071$, *p_S_* = 0.184). KBV was less prevalent and detected from April until June in hives from two sites that had Argentine ants (figure 2*c*). BQCV, DWV and KBV were found in all ant colonies in January and showed large variation in titres throughout the experiment (electronic supplementary material, figure S4).

In bees, the microsporidian *N. ceranae* and trypanosomatid *L. passim,* showed high prevalence in January and infection levels were not affected by Argentine ants (figure 2*d,e*, *N. ceranae*: *F_T_* = 0.732, $R_{T}^{2}=0.009$, *p_T_* = 0.451; *L. passim*: *F_T_* = 1.697, $R_{T}^{2}=0.018$, *p_T_* = 0.217). *Nosema ceranae* infection levels were also not affected by sampling site, time, nor the treatment–time interaction (*F_M_* = 0.558, $R_{M}^{2}=0.036$, *p_M_* = 0.567; *F_S_* = 0.890, $R_{S}^{2}=0.046$, *p_S_* = 0.489; *F_T × M_* = 1.492, $R_{T\times M}^{2}=0.096$, *p_T × M_* = 0.094). Infections levels of *L. passim* differed between sampling sites and time points (*F_M_* = 0.821, $R_{\mathrm{Time}}^{2}=0.043$, *p_M_* = 0.005; *F_S_* = 4.136, $R_{S}^{2}=0.172$, *p_S_* = 0.006) with no significant interaction (*F_T × M_* = 2.120, $R_{T\times M}^{2}=0.111$, *p_T × M_* = 0.066).

Using RNA-seq in bees collected six months after the start of the experiment, we detected 10 viruses that are associated with bees and none that were associated with ants. No significant shifts in the virome were found (electronic supplementary material, table S3, and figures S5 and S6).

## 4. Discussion

Invasive species including Argentine ants are well known for their negative influence on biodiversity through competition, predation and the formation of mutualistic interactions [30,31]. As multiple studies have linked ants to pathogens of pollinators [20,22,25,27] and beekeepers report hive losses owing to ants [40], our aim was to ascertain if this invasive ant could also affect pollinator pathogen dynamics. We show that virus titres including those of DWV, a virus linked to the death of millions of beehives around the globe [13,14], increased in honey bees when invasive Argentine ants were present.

Increases in BQCV and DWV titres in bees could indicate a viral spillback from Argentine ants. Ants are known to vector pathogens, such as human pathogens in hospitals [41] and kitchens [42]. Argentine ants could acquire viruses when entering infected beehives [27] and disperse them within hives and apiaries. Whether these ants are biological vectors, in which viruses are amplified or mechanical vectors that facilitate transmission without active viral replication [22] should be established. Evidence for DWV and KBV replication has been found in a range of non-*Apis* hosts [43] including Argentine ants [20,44]. The symptomatic wing deformities of DWV have also been observed in infected bumblebees [45] and hornets [46], further indicating its broad host range.

Bee viruses were present in ants before beehives were moved close to their nests, suggesting that infections are persisting in ants without bee presence. The prevalence and titres of some bee-associated viruses in ants, however, have been found to decrease outside apiaries [27,44]. KBV could be a more generalist insect pathogen that has repeatedly been found as part of the Argentine ant virome [47,48]. Beehives in our experiment were not infected with KBV until autumn, even though in ant-infested apiaries they were probably exposed to KBV-infected ants. Perhaps the accumulated exposure resulted in infections in bees. Contrary to the viral pathogens, the more specialized bee-microsporidian and trypanosomatid infections followed typical seasonal dynamics [49,50] and did not change with ant presence.

The interaction between bees and ants may have several detrimental effects on bees. An increased foraging effort as indicated by more returning foragers in hives with ants may be driven by higher demand for food to compensate for resources lost to ants. Increased foraging also increases the chances of bee exposure to pathogens from outside the hive. Accumulating stressors and their interactions, in particular between pathogens, has been shown to accelerate bee mortality [9]. The elevated DWV titres we found here could suggest that ants are an additional stress factor; the hives under ant attack appeared visibly distressed with bees that were attempting to defend their colony. Consequently, the interplay between virus and ant stress may affect bee immunity.

Although viral infections in honey bees have been extensively studied, gaps in our understanding of how interactions with other species affect bee disease remain. Argentine ants are expanding their range [51], so that their impact on bee pathogen dynamics may increase, perhaps even affecting the wider pollinator communities via viral spillover [52]. Our work suggests that controlling ant invasions could improve bee health and with it crop pollination. The links between DWV and Argentine ant presence need to be studied further to test the direction of transmission and the potential for DWV replication in ants in laboratory settings, where the detection of replicative DWV from ingested bee tissue can be excluded.

Our results indicate that an invasive species, which can reach extremely high densities over massive scales in its introduced range [30], can alter pathogen dynamics in honey bees. Other researchers have similarly found introduced species to alter pathogen dynamics of communities, particularly where the introduced species attains high abundance [52]. We suspect that many other introduced predators or competitors have similar ‘hidden’ effects in their introduced communities.

## Data Availability

The data and scripts that support the findings of this study are openly available from the Dryad Digital Repository: https://doi.org/10.5061/dryad.v15dv420f [37]. The data are provided in the electronic supplementary material [53].
